# Supplementary figures and images for: Discovering New QTNs and Candidate Genes Associated with Rice-Grain-Related Traits within a Collection of Northeast Core Set and Rice Landraces
Source: Plants (Basel). 2024 Jun 19;13(12):1707. doi: 10.3390/plants13121707 (PMC11207502; doi:10.3390/plants13121707)

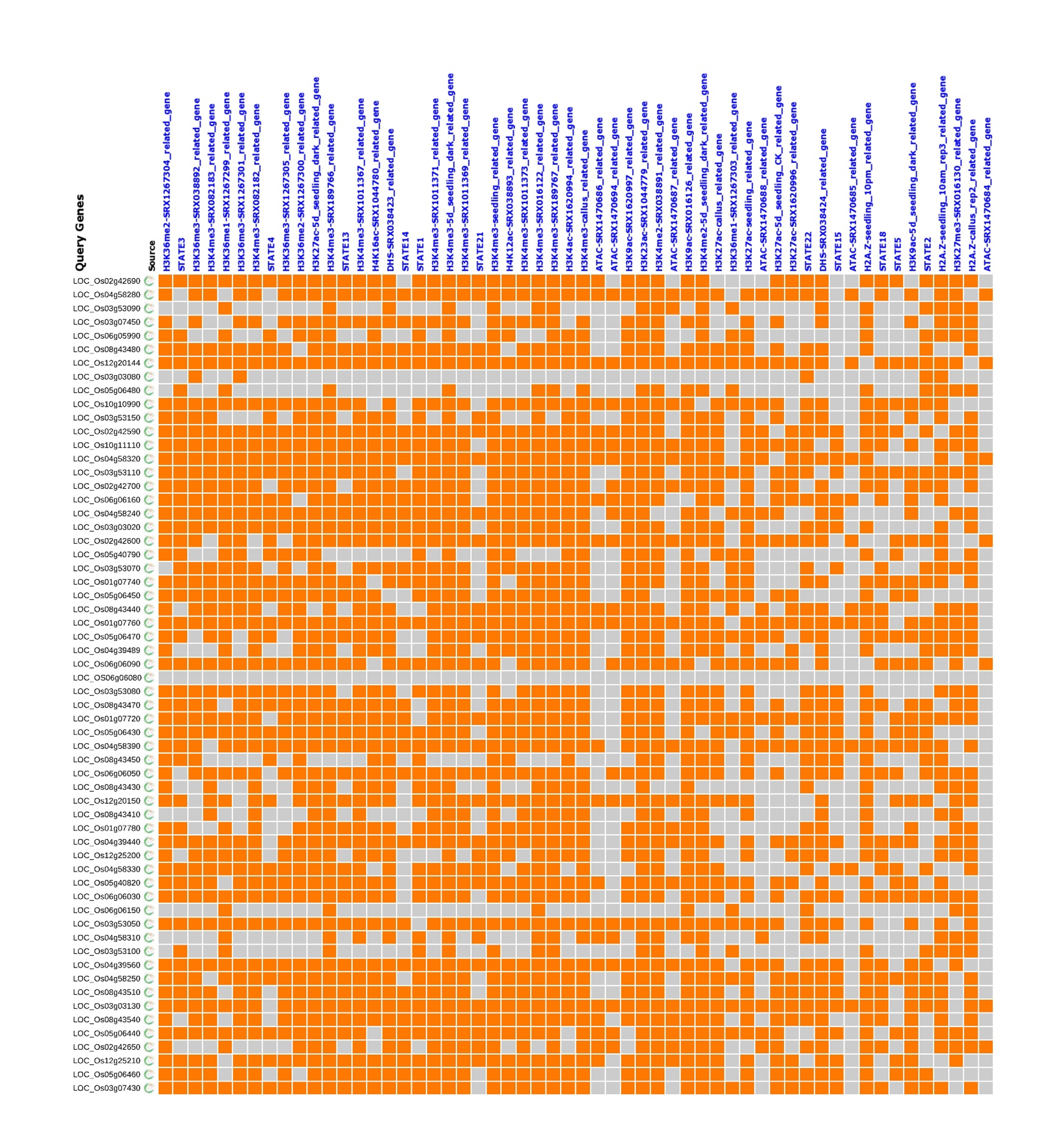

Supplement: Supplementary file 1 [file plants-13-01707-s001.zip › Supplementary Figures/Figure S3. Heat map of chromatin states of 60 candidate genes..jpg]
